# PEG-penetrated chitosan–alginate co-polysaccharide-based partially and fully cross-linked hydrogels as ECM mimic for tissue engineering applications

**DOI:** 10.1007/s40204-015-0041-3

**Published:** 2015-09-15

**Authors:** Anitha Radhakrishnan, Geena Mariya Jose, Muraleedhara Kurup

**Affiliations:** Department of Biochemistry, University of Kerala, Karyavattom, Thiruvananthapuram, Kerala India

**Keywords:** Tissue engineering, Cross-linked hydrogels, Alginate, Chitosan, ECM mimic

## Abstract

The emerging strategy of tissue engineering for the management of end-stage organ failure and associated complications mainly relies on ECM mimicking scaffolds for neo-tissue genesis. In the current study, novel polyethylene glycol interpenetrated cross-linked hydrogel scaffold based on a co-polysaccharide (PIAC) synthesized from two marine heteropolysaccharides, alginate and chitosan, was designed. Partially cross-linked (PIAC-P) and fully cross-linked hydrogels (PIAC-F) were prepared. The physiochemical evaluations of both the hydrogels revealed the presence of alginate fraction and extensive –OH groups on the surface, sufficient water content and water holding capacity. The porosity and bulk density were also appreciable. The scaffolds were hemocompatible and were able to adsorb appreciable plasma proteins on to the surface. MTT assay on hydrogel extracts and direct contact assay showed the nontoxic effects of fibroblast cells upon contact with the hydrogel. Live/dead assay using ethidium bromide/acridine orange cocktail on fibroblast cells grown on the hydrogels after 5 days of initial seeding displayed green nucleus revealing the non-apoptotic cells. PIAC-P hydrogels were superior to certain aspects due to the availability of free functional groups than PIAC-F where most of these groups were utilized for cross-linking. The biological evaluations confirmed the healthy being and 3D growth of fibroblasts on the porous networks of both the hydrogels. The present hydrogel can form an ECM mimic and can form a potent candidate for various tissue engineering applications.

## Introduction

The cells in a tissue are held together by colloidal extracellular matrix (ECM), which is gel like in consistency. The ECM component of the tissues forms an insoluble scaffold, which determines the shape and dimension of the organs (Cox and Erler 2011). The ECM integrates molecular signals that synchronize the specific function of the tissues on responding to the physiological demands. Moreover, the ECM also provides the structural and biological cues that aid in the proper alignment of cells to form a 3D organization (Even-Ram et al. 2006; Watt and Hogan 2000). This 3D microenvironment of the cells provided by native ECM enables them to function as a single unit. The diffusional exchange of oxygen and metabolites along the nanotopology of the ECM maintains the healthy being of the cells. The highly dynamic nature of the ECM causes several alterations during diseased conditions that result in the loss of cells specified for the desired function (Cattell et al. 1996).

An approach of direct application of cells to the injury site has been tried to restore the ECM structure and function. But only 10 % were found to be attached and were worn out immediately without survival. The cell death has occurred mainly due to the absence of 3D microarchitecture and associated inflammation (Hofmann et al. 2005). Several organ-assisted devices like heart–lung bypass machines or dialysis units have been employed for supporting the whole diseased organ or a part of it. All these devices could provide only temporary support, and the complete resumption of organ functions cannot be achieved (Thomson et al. 1995). The infections associated with these devices are also a common challenge (Ishaug et al. 1997). So the need of ECM substitutes that hold the cells until the rejuvenation of host tissue, post-disease stages, is highly demanding. An ECM substitute should act as a scaffold to carry the cells until a functional tissue is reestablished (Finosh and Jayabalan 2012). Such a scaffold can be made from synthetic and natural molecules or a combination of both, which can be effectively used for the ex vivo engineering of various organ parts. These scaffolds should be biocompatible, biodegradable and porous and support the normal proliferation and functioning of the cells seeded on to it (Gnanaprakasam Thankam et al. 2013). Incorporation of physiochemical, biological and mechanical cues along with these scaffolds improves the quality and durability of the ECM substitutes.

From a tissue engineering (TE) standpoint, the construction of a native ECM mimic for a specific application is crucial. But the complexity of ECM and cell–ECM interactions is challenging. An ideal TE ECM mimic should promote the attachment of specific cell types and stimulate the synthesis of their own ECM by themselves (Liu and Ma 2004). A simple scaffold material supporting cell growth and function with appreciable physiochemical, mechanical and biological properties is ideal for the in vitro construction of an ECM mimic. This mimic should be able to facilitate effective nutrient transfer, gas exchange, metabolic waste removal and signal transduction to and from the surrounding medium (Mikos et al. 1994). Several types of both natural and synthetic polymers have been employed for the synthesis of a suitable scaffold for the cell growth (Drury and Mooney 2003).

The hydrogel subset of biomaterials has gained prior significance to various TE applications due to their close similarities with native ECM (Peppas et al. 2006). Hydrogels were reported to be excellent for the in vitro construction of tissues and their 3D growth (Burdick and Vunjak-Novakovic 2009). The polymer segments in hydrogels are highly hydrophilic and can absorb a large amount of water due to the presence of interconnected microscopic pores. Porosity of the hydrogels facilitates potent mass transfer abilities and provides sufficient room for cell homing, which is very significant for the growth and infiltration of cells (Peppas et al. 2000). Furthermore, the hydrogels facilitate the deposition of ECM components to form the neo-organ as they degrade (Camci-Unal et al. 2014). Apart from these qualities, the biocompatibility offered by the hydrogels has added extra advantage to this class of biomaterials.

Brown sea algae-derived heteropolysaccharide alginate has been used as ECM mimic for various TE applications due to their greater hydration and low toxicity. Alginate is a hydrophilic polyanionic heteropolysaccharide comprising (1–4)-linked β-D-mannuronic(M) and α-L-guluronic (G) monomers (Gnanaprakasam Thankam and Muthu 2013). Chitosan is another marine heteropolysaccharide derived from the exoskeletons of mollusks and crustaceans which has been reported to be useful for various biomedical applications. The water holding capacity and biocompatibility of the chitosan make it an ideal material for drug delivery and the in vitro engineering of various organ parts (Wu et al. 2007). Polyethylene glycol (PEG) is a highly compatible FDA-approved synthetic polymer, which has been used for cell encapsulation, drug delivery, TE and several other biomedical applications. The presence of ample –OH groups and the subsequent hydrophilic character promotes their application as a TE scaffold (Stosich and Mao 2007). The physiochemical and biological evaluations of the mentioned polymers are apt for ECM mimic scaffolds for various TE applications (Levengood and Zhang 2014; Thankam and Muthu 2014a; Yu et al. 2013).

The reinforcement of synthetic polymers with natural ones has been proven to enhance the biological performance of the system, especially mechanical properties. For instance, the modification of chondroitin sulfate with PEG enhanced the mechanical integrity of the resultant mucoadhesive hydrogel without compromising the biological activity of chondroitin sulfate (Strehin et al. 2010). Another hybrid hydrogel scaffold based on covalently cross-linked heparin and star-shaped PEGs has been fabricated for application in neuronal cell replacement therapies. Heparin–PEG hydrogel forms a rigid network with tunable mechanical properties to accommodate different types of tissues. The PEG component acts as a suitable mechanical platform for bio-functionalization of heparin (Freudenberg et al. 2009). Recently a micro-engineered tissue construct gelatin methacrylamide was interpenetrated with PEG for obtaining adequate mechanical rigidity and to enable cell adhesion and encapsulation (Daniele et al. 2014). The mechanical fragility of hydroxyapatite-based bone scaffolds was also rectified by interpenetration with PEG. Impregnation of PEG has improved mechanical stiffness and porosity to the tissue construct and resulted in better cellular response (Pramanik et al. 2015). Similarly, Jiang et al. designed a composite hydrogel scaffold based on PEG and fibrin for vascular tissue regeneration where PEG forms a porous mechanical and structural template for the fibrin polymers to induce rapid vascularization (Jiang et al. 2013). Thus interpenetration with PEG has been proven to augment the biological activity of natural polymers and provide them with adjustable mechanical properties. The flexibility for chemical modification imparted by the PEG structure and its nontoxic nature can be effectively manipulated to fabricate mechanically stable biomimetic hybrid scaffolds based on natural polymers for TE applications.

Even though these natural polysaccharides possess excellent compatibility and biomimetic properties, their mechanical instability hinders their long-term applications. The interpenetration and reinforcement of these polymers with mechanically robust and biocompatible synthetic polymers are appreciable. Such a system can retain the beneficial characters of biological polymers for cell attachment, and the synthetic counterpart provides the desired mechanical strength. In order to address these issues, we co-polymerized alginate with chitosan and simultaneously interpenetrated the co-polysaccharide with PEG to form PEG-interpenetrated alginate–chitosan (PIAC) co-polysaccharide. The subsequent cross-linking of PIAC with Ca^2+^ and glutaraldehyde forms PIAC-based hydrogel scaffolds. The present hydrogels possess the beneficial physiochemical and biological responses of the individual polymers, which promote its application as an ideal ECM mimic.

## Materials and methods

### Materials

Sodium alginate (medium viscosity) from brown algae and chitosan (low molecular weight, degree of deacetylation 75–85 %) were obtained from Sigma-Aldrich (Spruce Street, St. Louis, USA). Polyethylene glycol 4000, disodium hydrogen phosphate, sodium chloride and calcium chloride were supplied by Merck specialities (Pvt. Ltd, Mumbai), India.

### Synthesis of PEG-interpenetrated alginate–chitosan co-polysaccharide (PIAC)

The PIAC co-polysaccharide was synthesized by the acid-catalyzed condensation of chitosan and alginate and PEG (Mw 4000) in the ratio 1:5:2.5, respectively. PEG was melted by heating at 60 °C and added two drops of conc. H_2_SO_4_. Then powdered alginate and chitosan were added and mixed thoroughly. The entire mixture was then allowed to set at room temperature. A hard solid mass of PIAC was formed which contained around 11.7 % chitosan, 58.9 % alginate and 29.4 % PEG. It was then dissolved in minimum distilled water (1 ml for 1 g PIAC) to form a viscous solution PIAC co-polysaccharide and stored at room temperature in an airtight container. That is, 1 g PIAC solution contains 5.85 % chitosan, 29.45 % alginate and 14.7 % PEG by weight.

### Fabrication of PIAC-based partially cross-linked and fully cross-linked hydrogels scaffolds

Two types of hydrogel scaffolds were fabricated from PIAC co-polysaccharide by solvent casting followed by freeze-drying. The alginate fraction of PIAC was cross-linked with Ca^2+^ to form partially cross-linked hydrogel scaffold which is designated as PIAC-P. Briefly, PIAC-P was synthesized by stirring 1 g PIAC along with 1 ml 2 % Na_2_HPO_4_ and 0.6 ml 2 % CaCl_2_ at 60 °C until uniform mixing was achieved. The mixture was then casted on a petri dish and incubated at 60 °C overnight. The hydrogel precursor sheet so formed was immersed in 10 % CaCl_2_ solution for 1 h for additional cross-linking. The cross-linked sheet was then washed with distilled water and freeze-dried to get the partially cross-linked PIAC-P hydrogel scaffold. Fully cross-linked PIAC-F hydrogel scaffold was prepared by adding 0.2 ml 1 % glutaraldehyde along with the mixture and proceeded as same as that of PIAC-P.

### Surface functional group analysis by ATR-IR analysis

ATR spectrum of the hydrogels was recorded by using Nicolet 5700 FTIR Spectrometer. Freeze-dried samples of PIAC-P and PIAC-F were used for recording the spectra.

### Surface morphology and average pore diameter

The surface morphology of freeze-dried hydrogels was imaged by environmental scanning electron microscopy (ESEM). From the images, the average pore diameter was calculated using the imaging software ImageJ 1.46r using the multi-measure plugin by randomly considering 20 pores.

### Determination of water content and holding capacity

The water content and water holding capacity and corresponding profiles of both the hydrogel scaffolds were determined by swelling in distilled water. The dry weights of six pieces (1 cm × 1 cm) each of the freeze-dried hydrogels were initially measured. Then these samples were immersed in distilled water for a period of 30 min. The swollen samples were wiped softly to clear away the surface water, and the weight was measured at 30 min interval until equilibrium was reached. The equilibrium water content (EWC) and weight swelling ratio of the hydrogels were determined as per published protocols (Finosh et al. 2015).

$$ {\text{EWC}} = \frac{{{\text{Swollen}}\;{\text{weight}} - {\text{dry weight}}}}{{{\text{Swollen}} \;{\text{weight}}}} \times 100 $$

$$ \% \; {\text{Swelling}} = \frac{{{\text{Swollen\,\,weight}} }}{\text{Dry\,\,weight}} \times 100 $$

### Measurement of porosity of hydrogels using ethanol replacement method

Freeze-dried hydrogels discs of known weights were immersed in absolute ethanol overnight and wet weight was measured. The porosity was then calculated using the equation (Gemeinhart et al. 2000).

$$ {\text{Porosity}} = \frac{{{\text{Wet}}\; {\text{weight}} - {\text{Dry}}\; {\text{weight}}}}{{\left( {\text{Density}} \right)\left( {\text{Volume}} \right)}} \times 100 $$

### Determination of apparent density

The hydrogels discs of known weight were soaked in distilled water overnight and the apparent density was calculated according to the equation (Nanda et al. 2013).

$$ \rho = \frac{{4\,{\text{m}}}}{{\pi d^{2} h}} $$

where is apparent density (g/cm^3^), is weight of scaffold swelling in water (gm), is the diameter of scaffold after swelling (cm), and *h* is the height after swelling (cm).

### Hemolysis assay

Two milliliters of blood was collected from healthy human volunteers in an anti-coagulating centrifuge tube and spun at 1500 rpm for 15 min to separate the plasma. The remaining RBC suspension was washed twice with physiological saline. All the hydrogel samples were extracted in sterile PBS for 48 h; 100 μl of this PBS was mixed with 100 μl dilute RBC suspension and incubated at 37 °C for 3 h. A +ve control was set up with 100 μl sterile distilled water and a –ve with 100 μl 0.9 % saline and both were mixed with equal volume of RBC suspension. After incubation, it was subjected to centrifugation at 3000 rpm for 10 min and the OD of the supernatant was determined at 541 nm. From the OD values, the % hemolysis was calculated (Thankam and Muthu 2014b).

### Red blood cell (RBC) aggregation assay

One milliliter of RBC (as prepared above) was diluted to 10 ml with saline. All the hydrogel scaffolds were extracted in 2 ml PBS for 48 h; 100 µl extract was added to 100 µl diluted RBC and incubated at 37 °C for 30 min and examined under bright-field microscope to evaluate RBC aggregation (Thankam and Muthu 2014b).

### Estimation of plasma protein adsorption on the surface of hydrogels

Four milliliters of blood was collected from healthy human volunteers into tubes containing heparin. The tubes were centrifuged at 1500 rpm for 10 min and the plasma was collected; 1 ml plasma was diluted to 10 ml with physiological saline and 1 ml of diluted plasma was added to the PBS swollen hydrogels and incubated for 2 h at 37 °C. Then the hydrogels were removed, and the protein fraction left out was determined by Lowry’s method using 1 mg/ml bovine serum albumin (BSA) as standard. From this the percentage adsorption was quantified (Gnanaprakasam Thankam and Muthu 2013).

In order to distinguish the types of plasma proteins adsorbed to the hydrogels, SDS-PAGE (sodium dodecyl sulfate—poly acrylamide gel electrophoresis) analysis was done. The PBS-swelled hydrogels were incubated with diluted plasma (1 in 9 ml PBS) for 1 h at 37 °C under shaking. Then the scaffolds were removed from the plasma solution, and the adsorbed protein fractions were removed. These samples were then subjected to SDS-PAGE analysis (10 % acrylamide) using previously published protocols (Gnanaprakasam Thankam and Muthu 2013). BSA and blood plasma were used as controls.

### Biological evaluation of hydrogels

L929 cells were used for biological evaluations. The cells were procured from NCCS, Pune, India, and maintained in DMEM containing 10 % fetal bovine serum and antibiotics in a humidified incubator with 5 % CO_2_.

### MTT assay using fibroblast cells

The scaffold samples (1 cm diameter) were autoclaved and used for studies. Initially, the extracts of all the hydrogels were prepared by incubating the hydrogels in 2 ml of serum-supplemented DMEM at 37 °C in a CO_2_ incubator for 24 h. Monolayer culture of L929 mouse fibroblast cell was initiated at a density of 5 × 10^3^ cells per 24-well and incubated for 24 h. Following incubation, the cell culture media were aspirated from the monolayers and were replaced with the extract of the hydrogels. All cultures were incubated at 37 °C for 24 h in a CO_2_ incubator. Then the culture was washed with PBS and 200 µl MTT per milliliter culture (MTT 5 mg/vol dissolved in PBS and filtered through a 0.2 µm filter before use) was added. The whole content was again incubated at 37 °C for 3 h and 300 µl DMSO was added to each culture well. Finally the whole content was incubated at room temperature for 30 min until all cells were lysed and a homogenous color was obtained. The solution was centrifuged for 2 min to sediment cell debris. The optical density (OD) was measured at 540 nm. Cells treated with MTT solution without hydrogel extract were used as control. Hydrogel treated with DMSO was used as blank. From the OD values, %viability was calculated (Thankam and Muthu 2014c), (Finosh and Jayabalan 2015).

### Direct contact assay

Hydrogel discs of 1 cm diameter were placed over a monolayer of L929 cells and the changes in cell morphology were microscopically evaluated (Olympus CKX41 with Optika Pro5 camera) (Thankam and Muthu 2014c).

### Live/dead assay

The fate of L929 fibroblasts in the hydrogels was determined by live/dead assay using ethidium bromide (EtBr) and acridine orange (AO) as per the previously published protocols (Thankam and Muthu 2014c). Around 2 × 10^5^ cells were seeded onto the hydrogels and allowed to grow for 5 days in DMEM supplemented with 10 % FBS. Then the hydrogels were washed twice in PBS and added 2 ml EtBr/AO mixture (1:1) to make a final concentration of 50 µg/ml for each dye. After addition, the excess dye was washed with PBS and immediately observed under a fluorescent microscope connected to a CMOS camera attached to a computer (Thankam and Muthu 2014a).

### Statistical analysis

All experiments consisted of six samples from each group. The values are presented as means ± standard deviations. Statistical analysis was done with one-way ANOVA using online calculator, Statistics Calculator version-3 beta, and the level of significance was set at *p* value <0.05.

## Results and discussion

### Synthesis and characterization of PIAC hydrogels

PIAC co-polysaccharide-based hydrogel scaffolds were synthesized employing acidic condensation reactions of secondary –OH groups of chitosan and the –COOH groups of mannuronic acid residues of alginate. An ester group was introduced as a result of condensation by the removal of a water moiety. PEG moieties were interpenetrated throughout the co-polysaccharide network by thorough mixing under warm conditions. PIAC was then cross-linked to form PIAC-based hydrogel scaffolds. The guluronic acid residues of the alginate fraction in the PIAC were subjected to chelation with Ca^2+^ ions to form the partially cross-linked hydrogel scaffold PIAC-P. The available –OH groups and –NH_2_ groups of PIAC-P were then cross-linked with glutaraldehyde to form fully cross-linked PIAC-F hydrogel scaffolds. The volume of chitosan in the system is lower when compared with that of alginate. So the interactions between the PEG and alginate will be more than that between chitosan and PEG. Still, the secondary –OH groups of the chitosan link to the –COOH groups of alginate through ester bonds for both the hydrogels. Also the free –NH_2_ groups of chitosan in the PIAC-F form Schiff base with the –CHO group of glutaraldehyde (cross-linking). The cross-linked glutaraldehyde is present in negligible quantities, and the unreacted glutaraldehyde is effectively cleared through extensive washing with water and culture medium.

**Fig. 1:**
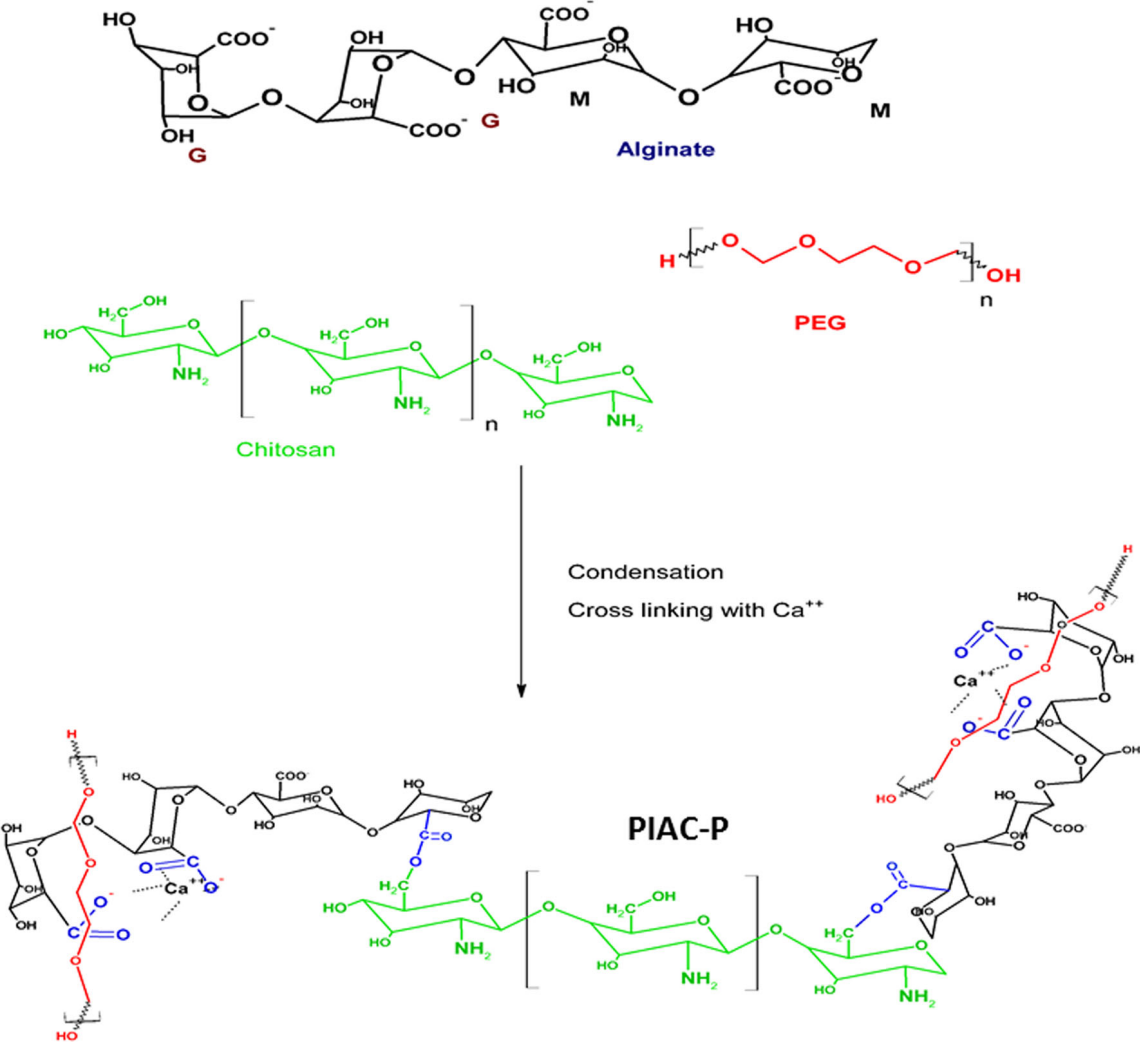
Synthesis of partially cross-linked PIAC-P hydrogel scaffolds

**Fig. 2:**
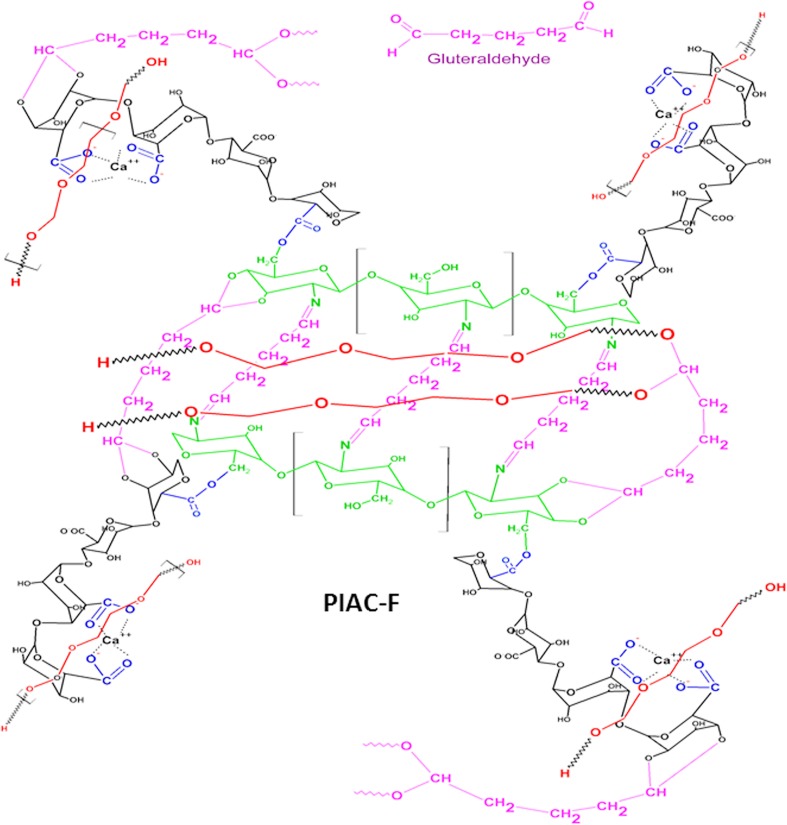
Synthesis of fully cross-linked PIAC-F hydrogel scaffolds

The interpenetrated PEG segments provide mechanical strength and hydrophilicity to the scaffolds. It has already been reported that the reinforcement/interpenetration of natural polymers with synthetic ones could improve mechanical properties and biological performance. Therefore, PEG, being biocompatible and slow degradable, can enhance the stability and durability of the system (Lohani et al. 2014; Dragan 2014). The details of the synthesis are given in Figs. 1 and 2.

Interpenetrating synthetic polymers into the hydrogel networks was reported to enhance their overall performance (Thankam and Muthu 2014c). Such hydrogels are typically prepared by synthesizing by swelling a hydrophilic polymer network in a second polymer/pre-polymer solution and polymerizing the latter to form water loaded network of two different polymers (Myung et al. 2008). Moreover, the weak mechanical properties of natural polysaccharides were found to be enhanced by co-polymerizing with synthetic polymers. The mechanical instability of alginate hydrogels was addressed by co-polymerization and subsequent vinyl cross-linking with unsaturated polyesters like poly propylene fumarate and poly mannitol-fumarate-co-sebacate. This approach has enhanced the overall performance of hydrogels without affecting the biocompatibility of the alginate (Finosh and Jayabalan 2015; Finosh et al. 2015; Gnanaprakasam Thankam and Muthu 2014). In the present study, we tried a different route, by co-polymerizing two polysaccharides in the presence of PEG. By choosing this route, we could achieve a proper entanglement of both synthetic and biological polymers. Moreover the mechanical fragility of conventional hydrogels due to abundant water content was overcome by interpenetration. Additional strength was imparted by cross-linking also. This was evident from the stability of the hydrogels in cell culture medium (DMEM) and PBS. Owing to their biocompatibility, the PEG-interpenetrated hydrogels can form ideal ECM mimic scaffold even for load-bearing tissues (Gong et al. 2003). We tried several ratios for the combination of the polysaccharides and PEG for the synthesis of PIAC- and PIAC-based hydrogel scaffolds. Our intention was to prepare a hydrogel scaffold sheet under controlled gelling conditions. The ratio was then optimized to be 1:5:2.5, respectively, for chitosan, alginate and PEG. Similarly the cross-linkers, Ca^2+^ ions and glutaraldehyde, were tuned for avoiding the cracking and breaking of the hydrogels.

### Physiochemical characterization

**Fig. 3:**
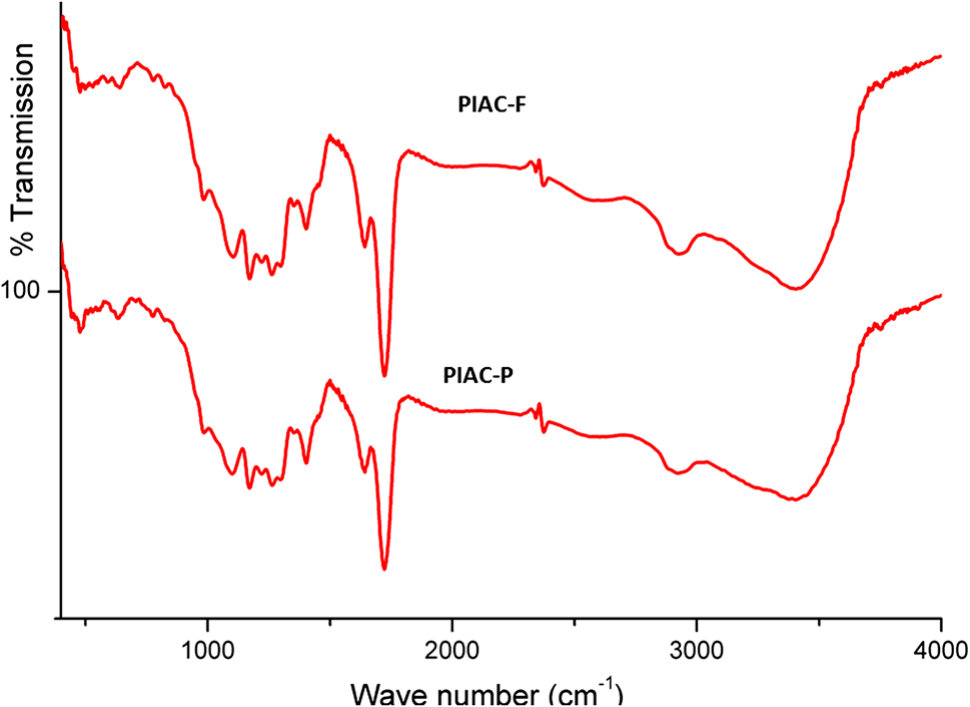
IR spectral analysis of PIAC-P- and PIAC-F-based hydrogel scaffolds

ATR spectral analysis showed the surface functionalities of PIAC-based hydrogel scaffolds as indicated in Fig. 3. The peak observed around 1600 cm^−1^ for carboxylate groups reveals the presence of calcium alginate units on the surface PIAC-P and PIAC-F. The broad peak at around 3400 cm^−1^ reveals ample amount of hydrogen-bonded hydroxyl groups imparted by PEG, alginate and chitosan. The peak at 1720 cm^−1^ for the C = O group stretching indicated the ester bond formation between alginate and chitosan. The peaks around 1100 cm^−1^ can be attributed to the C–O–C stretching of the alginate and chitosan. This is due to the acetal formation after glutaraldehyde cross-linking with the terminal –OH groups of PEG and the primary –OH groups of the polysaccharide fractions. Therefore it was concluded that alginate fraction, chitosan fraction and PEG segments are present on the surface of the hydrogel scaffolds and effective cross-linking.

### Water profile of hydrogels

**Fig. 4:**
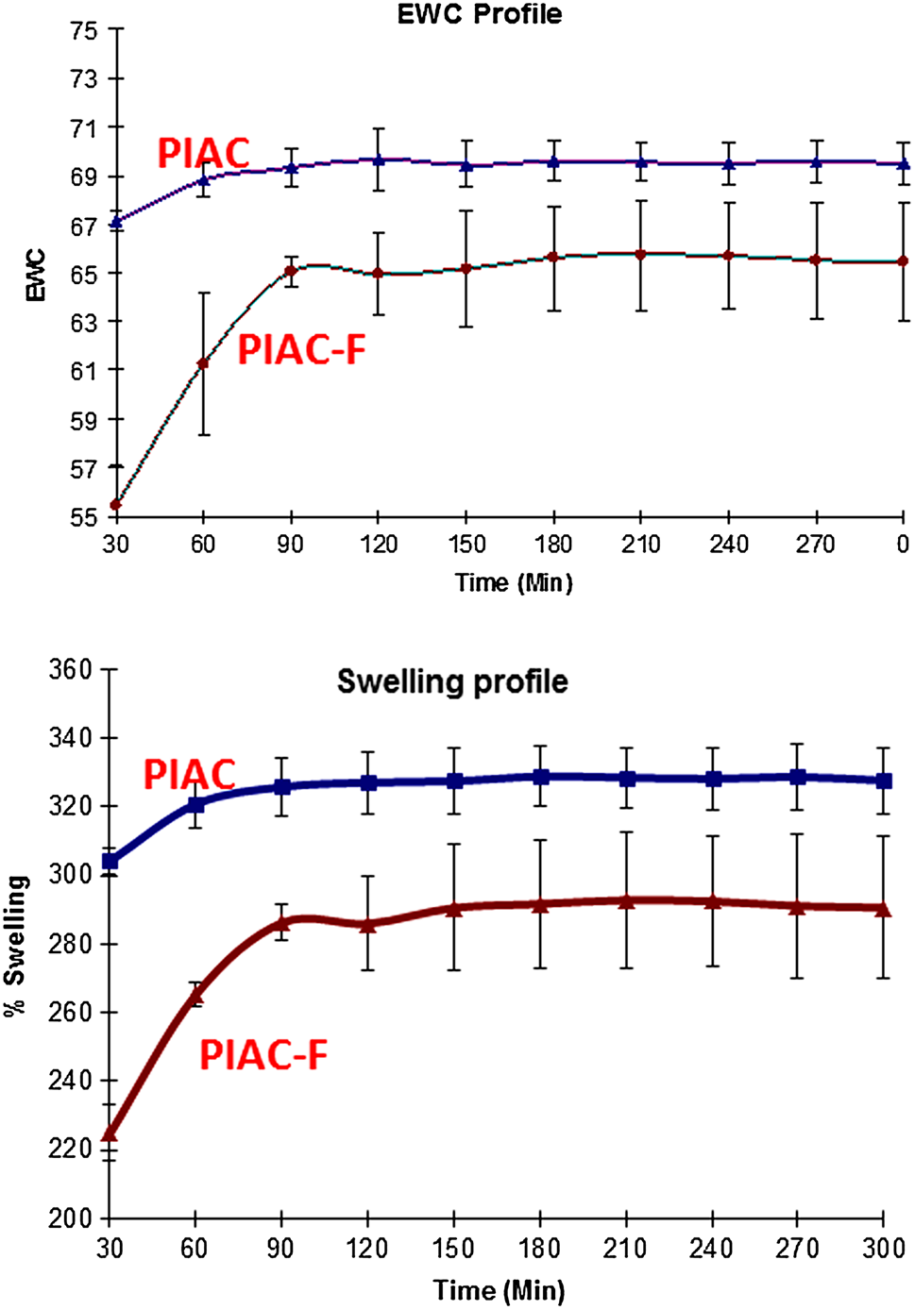
Water holding capacity and profile of PIAC-P-and PIAC-F-based hydrogel scaffolds. The values of time zero indicate the dry weights of the scaffolds

The EWC and water holding capacity of hydrogel are essential for the effective transport of water, nutrients and waste products between the cells and medium. The water profiling of the hydrogel showed a progressive increase in water absorption, and around 90 min an equilibrium value was attained. After 90 min, no further increase in swelling and EWC was observed as shown in Fig. 4. The water content and holding capacity after equilibrium swelling of PIAC are appreciable for supporting the cell growth and ECM deposition as given in Table 1. The swelling of hydrogels is a balance between contrasting osmotic forces and dispersing forces. The osmotic forces promote the swelling process by solvating the polymer functional groups, while the dispersing forces have an antagonistic effect. The dispersing forces largely depend on extend of cross-linking of the polymer networks (Gnanaprakasam Thankam et al. 2013). The relative lower values for swelling and EWC of PIAC-F were due to the additional cross-linking imparted by glutaraldehyde which was absent in PIAC-P.

**Table 1:** Evaluation of hydrogel scaffolds

Parameters (*n* = 6)	PIAC-P	PIAC-F
Swelling (%) (*P* < 0.001)	342.14 ± 54.16	246.72 ± 37.44
EWC (*P* < 0.05)	70.03 ± 5.67	65.28 ± 2.32
Average pore length	17.47 ± 3.43	35.16 ± 9.61
Porosity (%)	20.18 ± 5.7	13.16 ± 4.19
Apparent density (g/cm^3^)	0.38 ± 0.06	0.34 ± 0.04
Hemolysis (%)	1.82 ± 0.07	1.60 ± 0.007
Protein adsorption (%)	30.38 ± 4.41	22.21 ± 3.90
Viability (%)	104.23 ± 2.83	100.98 ± 3.79

### Porosity and apparent density of PIAC-based hydrogels

The porosity is a function of total volume of pores present in the hydrogels. The % porosity of PIAC-F hydrogels was found to be lower when compared with the other. But there was no drastic difference in apparent density as given in Table 1. The decrease in porosity of PIAC-F was due to the cross-linking by glutaraldehyde. The glutaraldehyde cross-linking reduced the total porosity by forming acetal and Schiff base with the otherwise free –OH and –NH_2_ groups, respectively. The alteration in alignment of polymer chains as a result of cross-linking has reduced the free space inside the PIAC-F hydrogel scaffolds than PIAC-P leading to the reduction in porosity. But the relatively similar bulk density of both the hydrogels was an indication of uniform pore size, distribution and permeability. Apparent bulk density can influence the mechanical properties of the scaffolds. From the porosity determination and apparent bulk density calculation, it was confirmed that the glutaraldehyde has masked some of the free functional groups without much affecting the bulk properties of the PIAC-F hydrogels (Nanda et al. 2013).

The porosity and pore interconnectivity are vital for ECM mimic hydrogel scaffolds for TE. The trafficking of nutrients and biomolecules from the medium is effectively facilitated through the interconnecting pores until functional vascular system is established. Moreover the proper orientation of newly formed tissue is mediated by pores through a process called contact guidance. The rate and quality of ECM synthesis and deposition by the seeded cells are also mediated by the porosity of the scaffolds. Furthermore these pores can interlock in vivo with the surrounding native tissues and enhance integration with the host. And the porosity requirements vary among the cells and tissues of interest (Thankam and Muthu 2014c).

The mass transport and fluid movement is very vital for the survival and functioning of the cells grown on hydrogel scaffolds. The pore size and interconnectivity determine the flow of nutrients and metabolites to and from the surrounding medium. The pore size is the distance of the attachment site of the cells in the scaffold during initial seeding with respect to the scaffold–medium interface. The pore size also influences the organization and orientation of cells in later stages of growth (Khoda et al. 2013). So the pores should possess an optimum size according to the particular application of interest. The pore lengths measured on the freeze-dried hydrogels are given in Table 1. The pore length of PIAC-F was found to be greater than that of PIAC-P. The pores are created as a result of cross-linking and freeze-drying. The greater cross-linking of PIAC-F expels comparatively bigger ice crystals than PIAC-P. This leaves bigger pores after freeze-drying under vacuum conditions. We have calculated the pore length of freeze-dried hydrogels. Imbibing and swelling of hydrogels in the medium cause volume expansion and pore opening. These structural rearrangements of the polymer chains can enhance pore interconnectivity when compared to the dried ones. But there are reports signifying the extra-large pores may lead to fibrosis and hinders neovascularization, especially in the case of TE. Madden et al. reported that a pore length of 10–45 μm was optimal for the development of cardiac tissue and beyond that the chances of fibrosis are very high (Madden et al. 2010). The pore sizes of both our hydrogels were within this limit signifying that the pore aspect is suitable for TE applications.

### Hemocompatibility assessment on PIAC hydrogel scaffolds

**Fig. 5:**
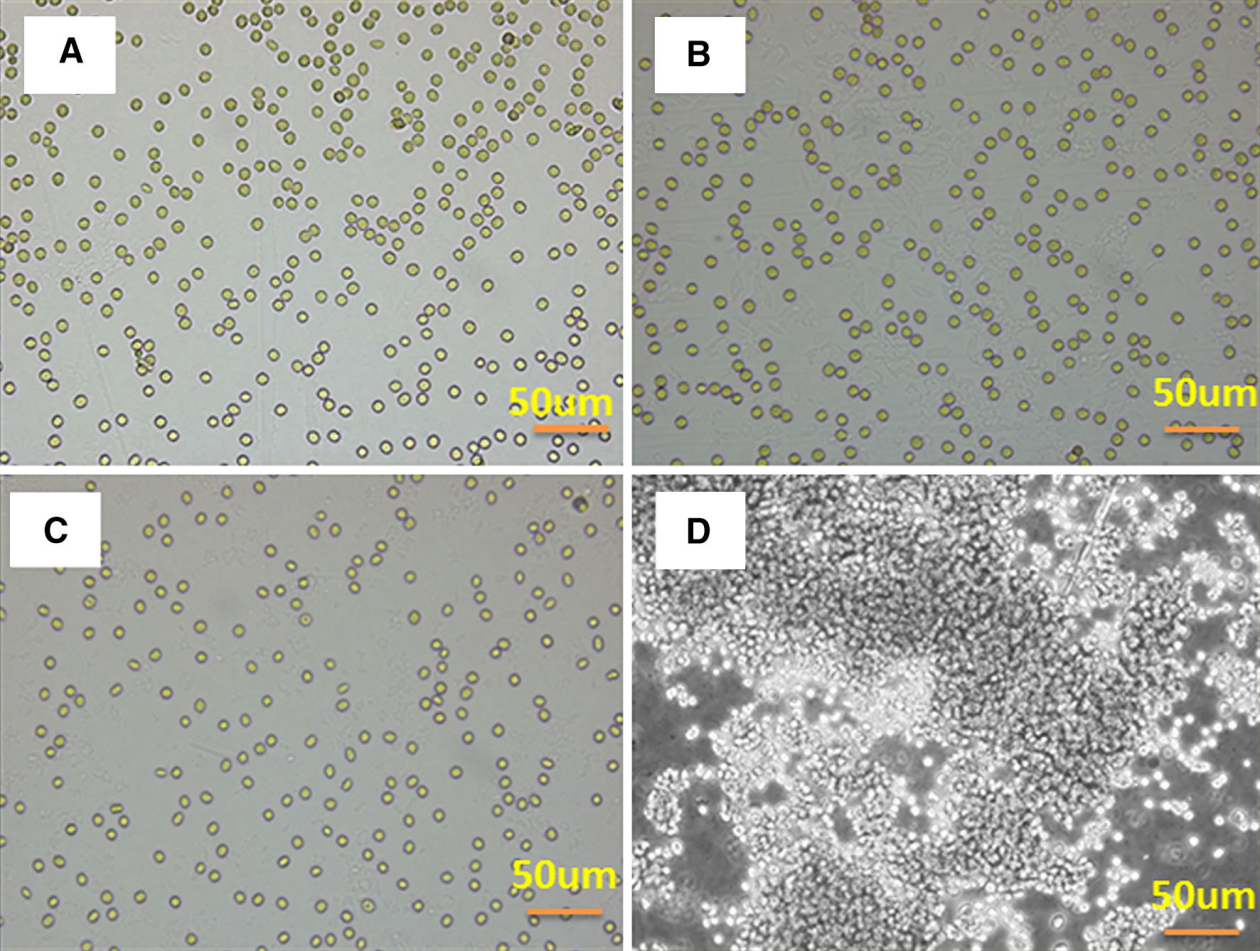
RBC aggregation studies showing the absence of aggregation in PIAC-P-based (**b**) and PIAC-F-based (**c**) hydrogel scaffolds in comparison with saline control (**a**) and positive control polyethyleneimine (**d**)

An ideal ECM mimic scaffold should not alter the normal rheology, physiology and the integrity of blood upon contact. The hemolytic potential of the hydrogel scaffolds is the measure of the extent of hemolysis that is induced by the hydrogel when it comes in contact with blood (Dawlee et al. 2005). Hemolysis assay on both the hydrogel extracts revealed that the hemolytic potential of our hydrogels was negligible and found to be within the acceptable limit of 5 % as in Table 1. Phase-contrast images of RBCs after incubation with the hydrogel extracts revealed the absence of aggregation as shown in Fig. 5. This was an indication of the absence of rouleaux formation of RBCs in contact with blood, showing that our hydrogel system has no repercussion on the fluidity and viscosity of blood. Moreover there occurs no unfavorable adsorption of macromolecules or leach out particles onto the membrane surfaces between erythrocytes.

Hemocompatibility is necessary for ECM mimics as these interact with blood directly or indirectly. The hemolysis gives the chances of RBC lysis upon contact with circulation. If hemolysis persists, it can further lead to anemia, jaundice and renal failure. A hemocompatible scaffold should be free from inducing thrombosis, thromboembolisms and antigenic responses (Qu et al. 2006). The obstruction in blood flow or changes in blood constituents cause the reversible aggregation of RBCs and leads to rouleaux formation. If the rouleaux persists, it will block the microcirculation too. RBC aggregation also occurs if there is any deformation in the membrane (Baskurt and Meiselman 1997). Hemocompatibility assays of both our scaffolds show their safer blood contacting application.

### Protein adsorption on PIAC hydrogel surface

**Fig. 6:**
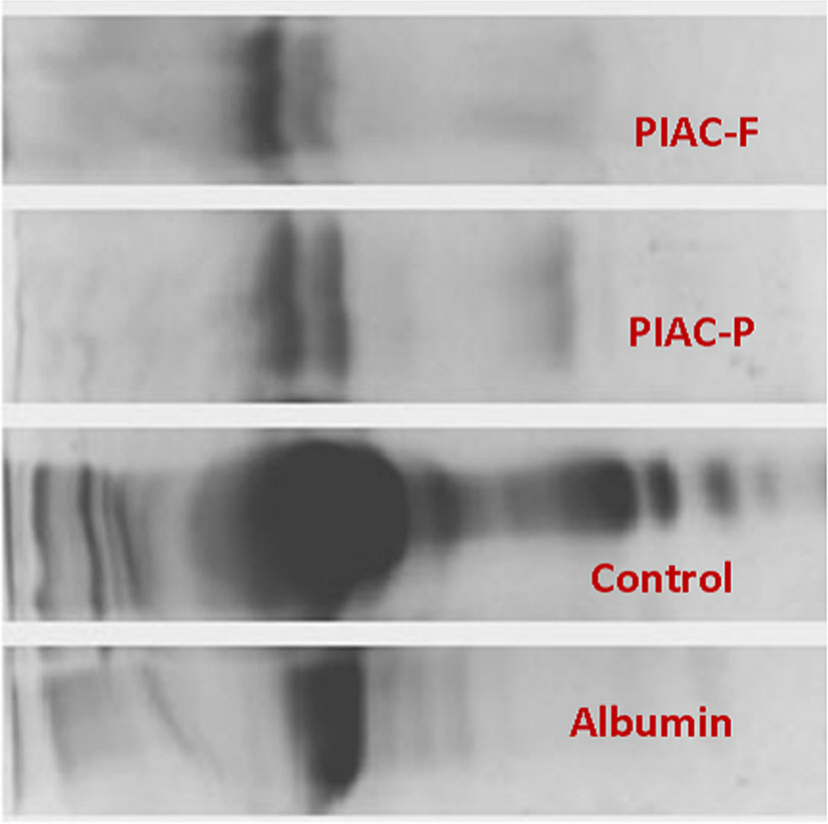
SDS-PAGE analysis of plasma protein adsorption on to PIAC-P- and PIAC-F-based hydrogel scaffolds showing thick bands corresponding to that of albumin

The total plasma proteins adsorbed on to the surface of PIAC-based hydrogels were found to be appreciable as given in Table 1. SDS-PAGE analysis revealed a thick band which corresponds to that of albumin indicating the considerable adsorption of albumin as shown in Fig. 6. The intensity of bands for albumin was comparatively lower in PIAC-F than PIAC-P. The relatively lower adsorption of PIAC-F can be attributed to their lower porosity and associated reduction in surface area due to cross-linking with glutaraldehyde. This reduction is also due to the lesser concentration of free functional groups in PIAC-F for the proteins to interact.

The plasma proteins adsorbed on the surface of hydrogel implants play a significant role in enhancing the biocompatibility of the material. Once the material is wetted with the water content of plasma, the adsorption of plasma proteins will occur immediately. The success of the implanted hydrogels is influenced by the amount and type of plasma proteins adsorbed on to their surface. Albumin and fibrinogen are the two major proteins of blood plasma. Upon adsorption on the biomaterials, they evoke contrasting responses. Albumin adsorption has a passivation effect that prevents thrombosis and subsequent inflammation reactions. But fibrinogen adsorption has an antagonistic effect to albumin. Still the adsorbed albumin layer can prevent fibrinogen binding (Gnanaprakasam Thankam and Muthu 2013). The predominant albumin adsorption on both the PIAC hydrogels enhances their compatibility to function as ECM mimics.

### Cellular compatibility of PIAC hydrogel scaffolds

**Fig. 7:**
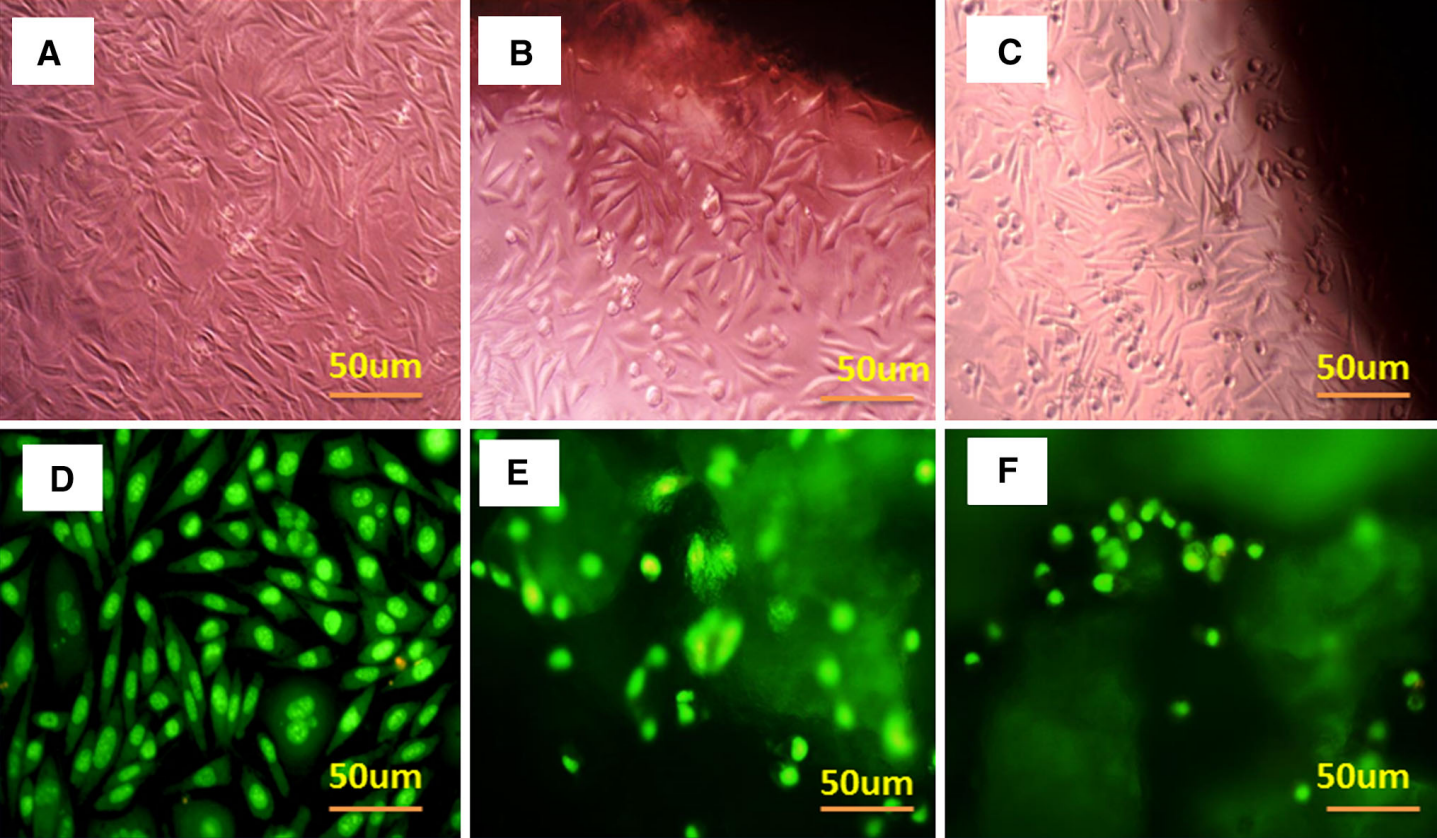
Cytocompatibility evaluations of hydrogels. Direct contact assay on L929 cells—control (**a**), PIAC-P-based (**b**) and PIAC-F-based (**c**) hydrogel scaffolds. Live/dead assay on L929 cells—control (**d**) PIAC-P-based (**e**) and PIAC-F-based (**f**) hydrogel scaffolds

In order to evaluate the cytocompatibility of the PIAC-based hydrogels, the percentage of viable cells on hydrogel extracts were quantified by the MTT cell viability assay. The particles leaching unreacted and degraded fragments were allowed to accumulate in the medium. The cell viability for both the scaffolds was found to be above 95 % as shown in Table 1. From this, it can be concluded that the presence of nontoxic degradation products or byproducts is minimal. The direct contact assay revealed that there was no change in cell morphology and viability of L929 cells grown on contact around the PIAC-based hydrogel as shown in Fig. 7. This again confirmed the absence of cytotoxicity and favorable compatibility of both PIAC-P and PIAC-F hydrogels to function as ECM mimic.

The live/dead assay showed mostly green fluorescence implying that the PIAC-based hydrogels can promote the survival of the seeded cells for long duration without inducing apoptosis as shown in Fig. 7. AO is a vital dye which will stain both live and dead cells. On the other hand, ethidium bromide (EtBr) can only enter the cells once their membrane integrity is lost. The live cells display a green nucleus, while the dead cells give orange fluorescence. The apoptotic or necrotic cell death will display a color ranging between green and orange depending on the stage of cell death. In short the live/dead assay revealed non-apoptotic and the healthy well-being of the cells on both the hydrogel networks signifying their ECM mimicking potential.

## Conclusions

The synthesized cross-linked hydrogels based on PIAC showed appreciable water holding and other physiochemical properties. The biocompatibility and the albumin passivation effect exhibited by both fully cross-linked (PIAC-F) and partially cross-linked (PIAC-P) hydrogels paved ways for their better cytocompatibility. Even though both the hydrogels are suitable for TE as scaffolds, PIAC-F predominated in terms of its greater stability imparted by glutaraldehyde cross-linking.
